# SUMOylation of DEC1 Protein Regulates Its Transcriptional Activity and Enhances Its Stability

**DOI:** 10.1371/journal.pone.0023046

**Published:** 2011-08-01

**Authors:** Yongde Hong, Xinrong Xing, Shujing Li, Hailian Bi, Chunhua Yang, Feng Zhao, Ying Liu, Xiang Ao, Alan K. Chang, Huijian Wu

**Affiliations:** School of Life Science and Biotechnology, Dalian University of Technology, Dalian, China; Cleveland Clinic, United States of America

## Abstract

Differentiated embryo-chondrocyte expressed gene 1 (DEC1, also known as sharp2, stra13, or BHLHB2) is a mammalian basic helix-loop-helix protein that is involved in many aspects of gene regulation through acting as a transcription factor. Changes in DEC1 expression levels have been implicated in the development of cancers. Using COS-7 cell, we showed that DEC1 can be modified by the small ubiquitin-like modifiers, SUMO1, 2 and 3. Two major SUMOylation sites (K^159^ and K^279^) were identified in the C-terminal domain of DEC1. Substitution of either K^159^ or K^279^ with arginine reduced DEC1 SUMOylation, but substitution of both K^159^ and K^279^ abolished SUMOylation, and more protein appeared to be retained in the cytoplasm compared to wild-type DEC1. The expression of DEC1 was up-regulated after serum starvation as previously reported, but at the same time, serum starvation also led to more SUMOylation of DEC1. In MCF-7 cells SUMOylation also stabilized DEC1 through inhibiting its ubiquitination. Moreover, SUMOylation of DEC1 promoted its repression of CLOCK/BMAL1-mediated transcriptional activity through recruitment of histone deacetylase1. These findings suggested that posttranslational modification of DEC1 in the form of SUMOylation may serve as a key factor that regulates the function of DEC1 *in vivo*.

## Introduction

Differentiated embryo-chondrocyte expressed gene 1 (DEC1) and it's related protein DEC2, are members of the basic helix-loop-helix (bHLH) family proteins that are involved in a number of cell processes, including proliferation, apoptosis, and circadian rhythms [1]–[4]. DEC1 is a 45.5-kDa protein that is expressed in a number of human and mouse tissues, especially in skeletal muscle [5], [6]. DEC1 contains one bHLH domain in its N-terminal region and three α-helices in its C-terminal region [7]. Its sequence shows the highest homology with the class E members of bHLH family, which is classified into six phylogenetic groups, A–F. However, DEC1 lacks a WRPW motif in its C-terminus, which is essential for the recruitment of the co-repressor Groucho[8]. There is now a growing body of evidence suggesting that DEC1 works as a transcriptional repressor, participating in the transcriptional repression of peroxisome proliferator-activated receptor (PPAR) and phosphoenolpyruvate carboxykinase (PEPCK) [9], [10]. DEC1 can also act as a self-repressor by binding to E-box (CACGTG) via a histone deacetylase 1 (HDAC1)-dependent pathway or through interacting with components of the basal transcription machinery, such as the transcription factor IIB (TFIIB) and the TATA binding protein (TBP)[11]–[14]. In the mammalian clock system, DEC1 also serves as a repressor for CLOCK/BMAL1 heterodimer-mediated promoter activity, through recruitment of HDAC1 [15].

Recently, DEC1 was found to interact with nuclear receptors, including PPAR, liver X receptor (LXR) and retinoid X receptor (RXR) through its conserved LXXLL nuclear receptor-interacting motif (LKDLL) in the N-terminal region, suggesting that DEC1 mediates the ligand-dependent LXR signal and functions as a corepressor of RXR heterodimers [16], [17]. In addition, DEC1 expression has been shown to be either upregulated or downregulated in different tumors, such as breast cancer, nonsmall-cell lung cancer, gastric cancer, during development [18]–[22]. However, the important role that DEC1 plays in gene expression and the molecular mechanisms underlying its activity in the regulation of other transcription factors remain unclear.

SUMO (small ubiquitin-like modifier) is a small protein that is covalently attached to its substrate at a lysine residue in the sequence ψKXE, where ψ is a large hydrophobic amino acid and X represents any amino acid. SUMO is cleaved from the SUMO-protein conjugate by SUMO-specific proteases (SENPs) [23], [24]. Although, the sequence of SUMO is distantly related to ubiquitin (18% identity), the two proteins have similar three-dimensional structures. Interestingly, SUMO has an unstructured amino-terminal extension containing 22 residues that it not found in ubiquitin, and this may provide an additional signal sequence for prospective interacting proteins [25], [26]. The SUMO conjugation pathway and the enzymes involved in the pathway have striking similarities to those of ubiquitination. However, it has been found that modification by SUMO has a range of functions that is different from ubiquitin (which mainly targets its substrates for degradation) including regulations of protein-protein interaction and transcription, alteration of subcellular localization of proteins, and DNA repair [27]–[29]. A large number of proteins have been discovered that are subjected to SUMOylation, and many of them are nuclear proteins with important roles in cell processes. Since DEC1 has two consensus SUMO acceptor sites, K^159^ and K^279^, both of which are contained within the ψKXE sequence, we wanted to investigate whether DEC1 can be modified by SUMO.

In this report, we showed that DEC1 can be modified by SUMO1, 2 and 3 and demonstrated that SUMOylation occurred at K^159^ and K^279^ of DEC1. Since DEC1 is associated with breast cancer development, we further investigated how SUMOylation may affect its activity in MCF-7 cells. We chose the core of clock protein, CLOCK, which is involved in the regulation of breast cancer development and is known to up-regulate DEC1 expression, and examined whether such interaction is affected by SUMOylation of DEC1 [30], [31]. Our results showed that SUMOylation of DEC1 enhanced its repression of CLOCK/BMAL1-mediated transcription activity through recruitment of HDAC1 in MCF-7 cells.

## Results

### DEC1 is modified by SUMO1, 2 and 3

**Figure 1:**
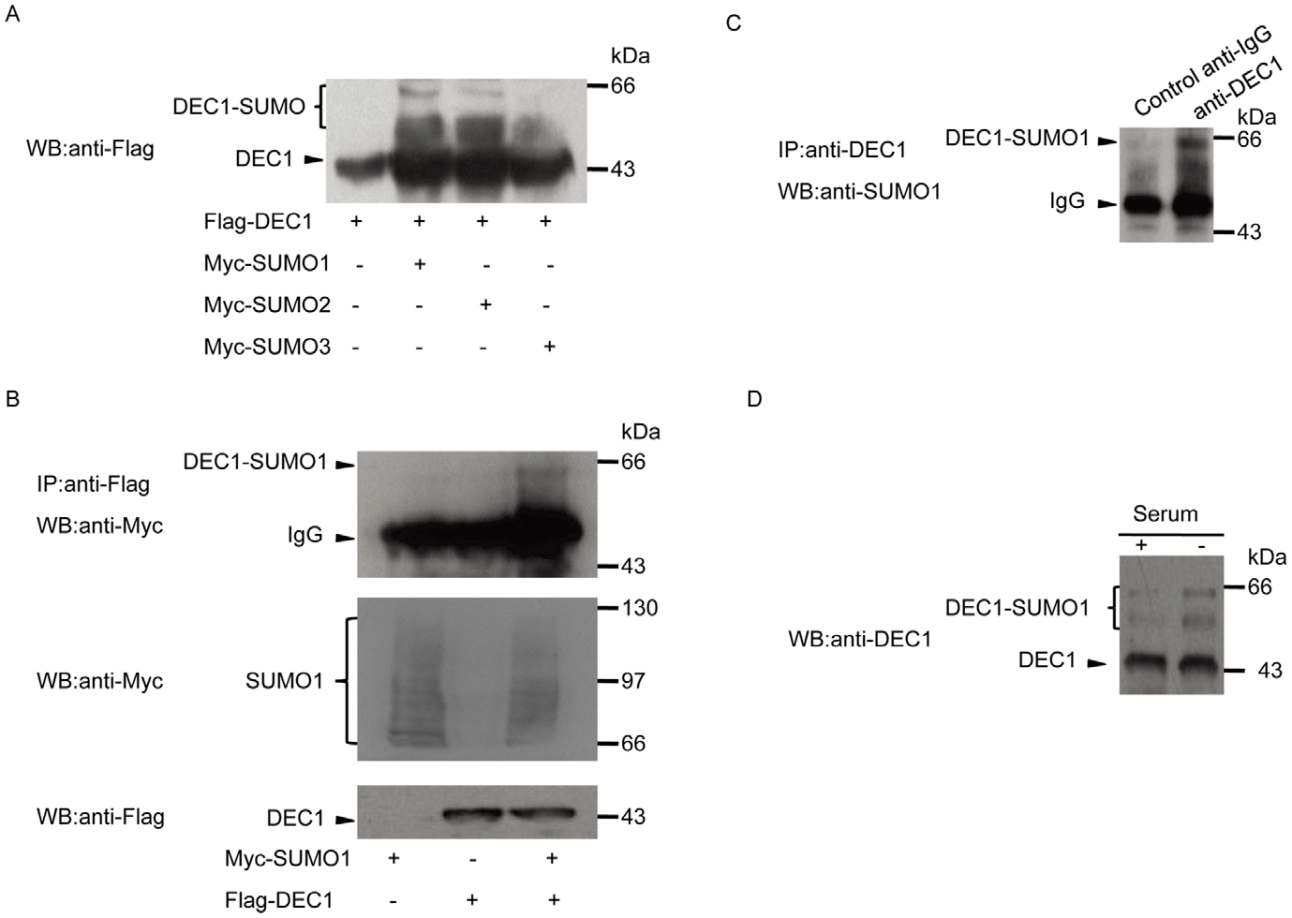
Modification of DEC1 by SUMO1, 2 and 3. (A) COS-7 cells were co-transfected with Myc-tagged SUMO1, 2 or 3 and Flag-tagged DEC1, and then subjected to western blot with anti-Flag antibody. (B) MCF-7 cells were co-transfected with Flag-tagged DEC1 and Myc-tagged SUMO1 and then subjected to immunoprecipitation with anti-Flag antibody followed by western blot with anti-Myc antibody. (C) Untransfected MCF-7 cells were subjected to immunoprecipitation with anti-DEC1 antibody or anti-IgG antibody followed by western blot using anti-SUMO1 antibody. (D) MCF-7cells were cultured in the presence of 10% serum for 24 h. The medium was then replaced with fresh medium containing either 10% or 0.8% serum and further incubated for 12 h, and the cells were finally subjected to western blot with anti-DEC1 antibody.

To determine whether DEC1 can be modified by SUMO, COS-7 cells were transfected with both Flag-tagged DEC1 and one of the three Myc-tagged SUMO expressing plasmids. Western blot analysis of the soluble fraction of cell lysate using anti-Flag antibody showed that DEC1 was efficiently modified by SUMO1, 2 and 3 (Figure 1A). Since the function of SUMO1 has been well characterized compared to SUMO2 and 3, and that DEC1 was mainly modified by SUMO1, we therefore mainly focused on the activity of DEC1 resulting from its interaction with SUMO1 in subsequent experiments. In addition, analysis of the SUMOylation of DEC1 in MCF-7 cells co-transfected with Flag-tagged DEC1 and Myc-tagged SUMO1 showed that over-expressed DEC1 was SUMOylated by SUMO1 (Figure 1B). Endogenous DEC1 in MCF-7 cells was also SUMOylated by SUMO1 (Figure 1C), and the level of SUMOylation was increased under serum starvation (Figure 1D). This result indicated that serum may regulate the SUMOylation of DEC1.

### DeSUMOylation of DEC1-SUMO conjugate is mediated by SENP1

**Figure 2:**
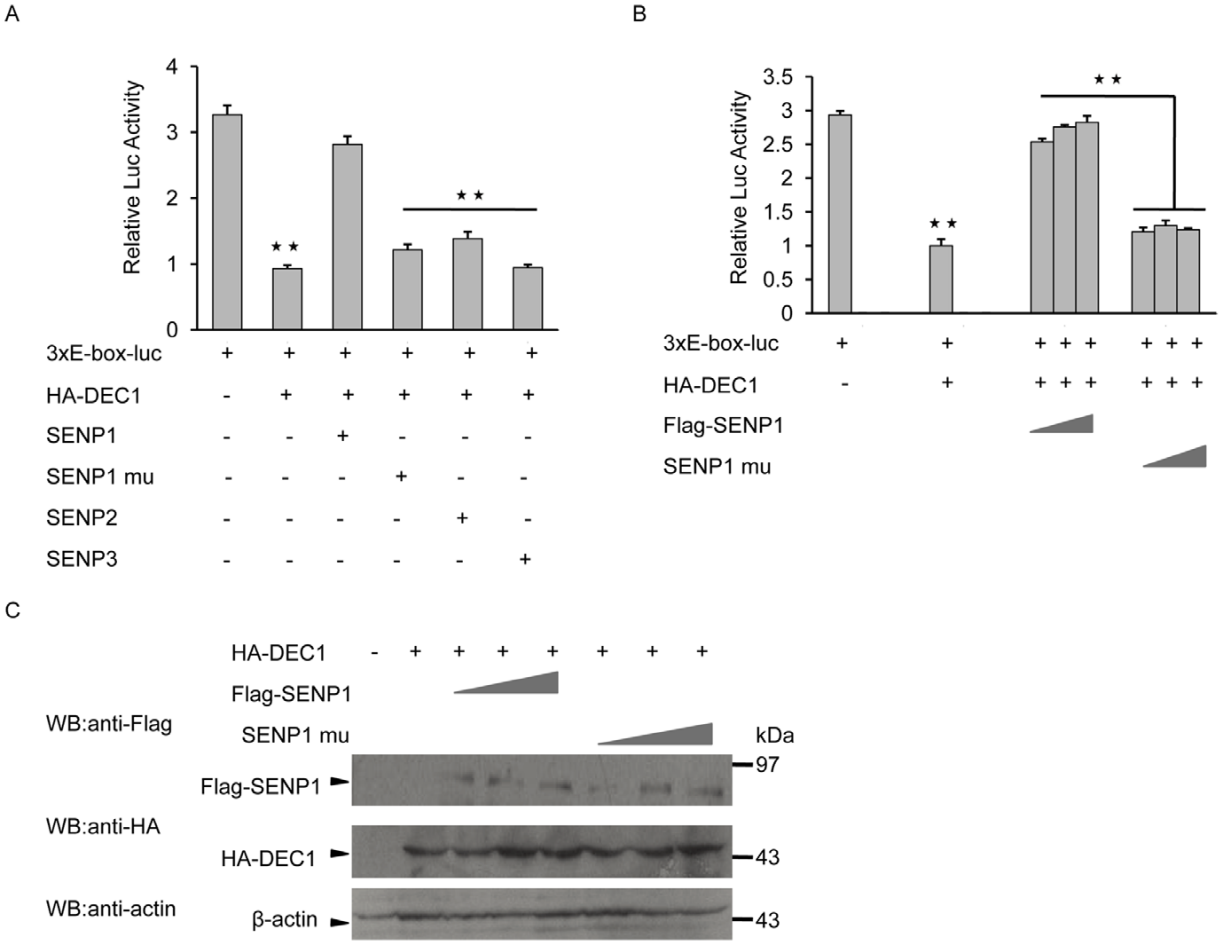
Sensitivity of DEC1 SUMOylation to SENP1. (A) COS-7 cells were transfected with HA-tagged DEC1 together with 3xE-box-luc reporter and either Flag-tagged SENP1, SENP1 mutant (mu), SENP2 or SENP3. Cells were then collected and subjected to luciferase activity assay. (B) COS-7 cells were transfected as in (A) but with increasing amounts of Flag-SENP1 or SENP1 mutant. (C) COS-7 cells transfected as in (B) were subjected to western blot with anti-Flag or anti-HA antibody. **, *p*<0.01

SUMOylation is a reversible process that is catalyzed by SUMO-specific proteases that cleave the isopeptide bond between SUMO and the target lysine residue. Three SUMO-specific proteases have been found in the nucleus, including SENP1, SENP2 and SENP3. In order to determine which SENP would affect the activity of DEC1, COS-7 cells were co-transfected with 3xE-box-luc, Flag-SENP1, SENP2 or SENP3 with HA-tagged DEC1 and the level of reporter activity in these cells was compared with that of COS-7 cells transfected with 3xE-box-luc only. The level of reporter gene activity of COS-7 cells co-transfected with 3xE-box-luc, DEC1 and SENP1 was almost three-fold that of COS-7 transfected with DEC1 only, but was almost similar to that of COS-7 cells transformed with only 3xE-box-luc (Figure 2A). COS-7 cells transfected with 3xE-box-luc and with or without SENP1 had similar levels of reporter gene activity, indicating that SENP1 by itself, did not influence the level of reporter activity (Data not shown). Compared to cells transfected with 3xE-box-luc and DEC1 only, moderate increases in reporter gene activity were also observed when the cells were co-transfected with 3xE-box-luc, DEC1 and either the SENP1 mutant R630L/K631M (which had lost its catalytic activity) or SENP2, while cells transfected with 3xE-box-luc and SENP3 showed no change in reporter gene activity. In addition, the effect of SENP1 on 3xE-box-luc activity was dose-dependent (Figure 2B). In all cases the expressions of HA-DEC1 and Flag-SENP1 were confirmed by Western blot (Figure 2C). This indicated that only SENP1 could affect the activity of DEC1 through deSUMOylation, and that SUMOylation of DEC1 significantly reduced the level of reporter gene activity.

### K159 and K279 are primary sites for the SUMOylation of DEC1

**Figure 3:**
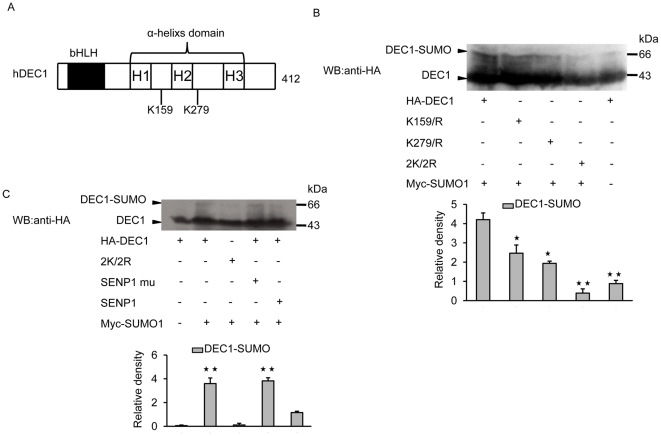
Identification of SUMOylation sites in DEC1. (A) Putative SUMOylation sites of DEC1. (B) COS-7 cells were transfected with HA-tagged wild-type DEC1 or its mutants K159R, K279R or K159R/K279R (2K/2R) and Myc-tagged SUMO1, and then subjected to western blot with anti-HA antibody. (C) COS-7 cells were transfected with HA-tagged wild-type DEC1 or its mutant K159R/K279R (2K/2R) with or without Flag-tagged SENP1 or SENP1 mutant (mu) and then subjected to western blot with anti-HA antibody. *, *p*<0.05; **, *p*<0.01.

Analysis of the human DEC1 amino-acid sequence revealed two conserved SUMO conjugation sites, AK^159^HE and IK^279^QE, in the C-terminal domain (Figure 3A). Furthermore, these two putative SUMOylation sites are conserved in different species, such as human, rat, zebra fish and mice. To test whether K^159^ and K^279^ are required for the SUMOylation of DEC1, COS-7 cells were co-transfected with either HA-tagged wild-type or mutant DEC1 along with SUMO1. Changing either K^159^ or K^279^ to arginine resulted in reduced sumoylation of DEC1, while changing both K^159^ and K^279^ to arginine led to further loss in SUMOylation of DEC1 (Figure 3B). The mutants K^159^R and K^279^R exhibited 42% and 54% reduction in SUMOylation, respectively, relative to wild-type DEC1, and while the band displayed by the double mutant 2K/2R could well be background signal, the band displayed by wild-type DEC1 in the absence of Myc-SUMO1 could have derived from endogenous SUMO (Figure 3B). This indicated that both K^159^ and K^279^ of DEC1 can be SUMOylated and substitution of either residue with arginine would result in significant reduction in SUMOylation. However, since SUMOylation was only abolished in the case of the double mutant 2K/2R, all subsequent experiments whereby comparison between wild type and mutant was made, only the double mutant was used. To prove that the high molecular weight band detected by western blot that corresponded to DEC1-SUMO was indeed SUMOylated DEC1, COS-7 cells were co-transfected with DEC1, SUMO1 and either SENP1 or its mutant. As expected, the level of SUMOylated DEC1 was also reduced to that of background level when the cells were co-transfected with SUMO1, wild-type DEC1and SENP1, but not its inactive mutant (SENP1 mu) (Figure 3C), thus further confirming the SUMOylation event of DEC1. Almost no SUMOylation of DEC1 was observed in the absence of over-expressed SUMO1 (Figure 3C).

### Effect of SUMOylation on nucleolar distribution of DEC1 and of serum starvation on DEC1 expression

**Figure 4:**
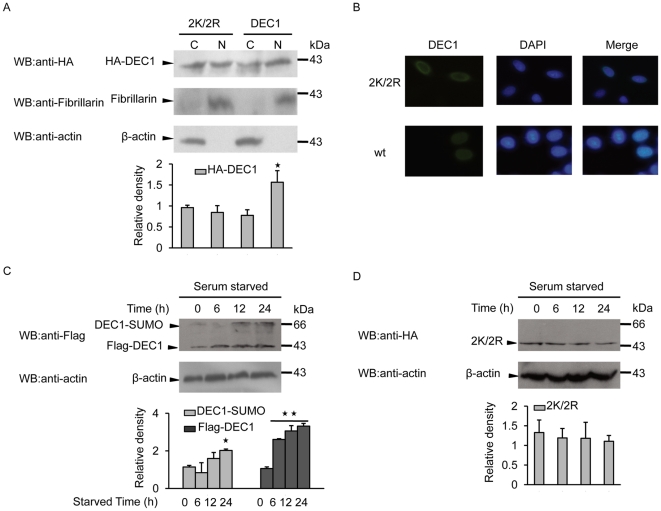
Effect of SUMOylation on nucleolar distribution of DEC1 and sensitivity of DEC1 SUMOylation to serum starvation. (A) MCF-7 cells were transfected with HA-tagged wild-type or mutant (mu) DEC1. Cytosolic and nuclear fractions were subjected to western blot using anti-HA antibody. Fibrillarin and β-actin were measured to monitor the efficiency of nuclear and cytosolic preparations, respectively. The graph shows the intensities of DEC1 bands in the cytosol and nucleus. (B) MCF-7 cells were stained with mouse anti-HA antibody (green) and then counterstained with DAPI (blue) for nucleus detection. MCF-7 cells were transfected with Flag-tagged wild-type DEC1 (C) or HA-tagged DEC1mutant 2K/2R (D) and grown in the presence of 10% FBS before were transferred to a medium containing 0.1% FBS. Cells were collected at the indicated times and subjected to western blot with the corresponding antibody. *, *p*<0.05; **, *p*<0.01.

DEC1 is predominantly localized in the nucleus, but can also be found in the cytoplasm. SUMOylation is known to affect the subcellular localization of a protein [32], [33]. To test whether SUMOylation influence the nucleolar translocation of DEC1, MCF-7 cells were transfected with HA-tagged wild-type DEC1 or its mutant 2K/2R and then subjected to nucleo-cytoplasmic separation. The mutant showed a reduced level of nucleolar protein compared to wild-type DEC1, and though the difference was not so prominent as seen from the blot, quantitative analysis of individual bands showed a reduction of at least 40%, while a slight increase of about 20% in cytoplasmic DEC1 was also obvious (Figure 4A). The increase in cytoplasmic level of DEC1 was somewhat much less than the corresponding reduction in nucleolar level in the case of the mutant. This may probably be due to loss of mutant protein via proteolysis brought about by enhanced protein instability. The lack of effect that SUMOylation had on the nucleolar level of DEC1 was also consistent with result obtained from immunofluorescence staining of the cells (Figure 4B). Previous study has reported that the level of DEC1 expression was increased under serum starvation [14]. To determine whether serum starvation can also affect the SUMOylation of DEC1, MCF-7 cells were transiently transfected with wild-type DEC1 and subjected to serum starvation for four different time periods. The result showed that the level of DEC1 protein was upregulated after 6 h of serum starvation, while no increase in the level of SUMOylated DEC1 was detected until after 12 h (Figure 4C), in which about 30% increase in SUMOylated DEC1 was observed, while the level of total DEC1 was more than two-fold compared to zero time. Prolonging the serum starvation time to 24 h led to further significant increases in both the levels of SUMO-DEC1 and total DEC1, becoming almost two-fold and three-fold, respectively, of those at zero time. SUMOylation of DEC1 was therefore sensitive to serum starvation and such sensitivity was dependent on time. In contrast, the mutant showed no increase in either the level of total protein or its corresponding SUMO-conjugate, while a slight drop in the level of total protein was obvious with increases in serum starvation time (Figure 4D).

### SUMOylation of DEC1 causes repression of CLOCK/BMAL1 activity

**Figure 5:**
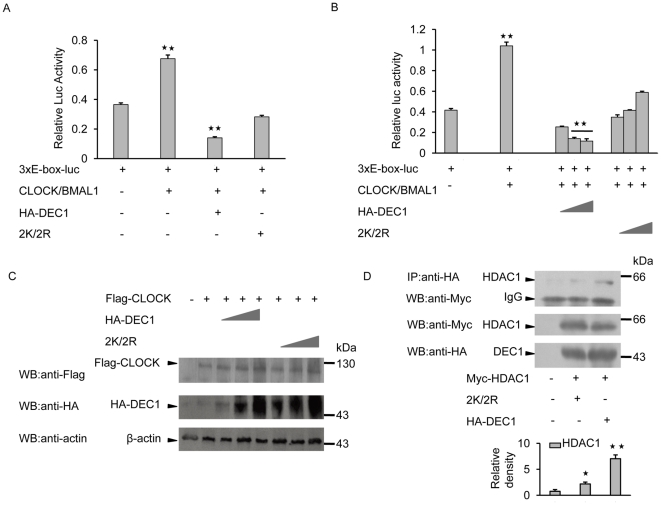
Effect of DEC1 SUMOylation on the repression of CLOCK/BMAL1 activity. (A) MCF-7 cells were transfected with HA-tagged wild-type DEC1 or its mutant 2K/2R with 3xE-box-luciferase reporter and Flag-tagged CLOCK/BMAL1 and luciferase activity was determined. (B) MCF-7 cells were transfected as in (A) but with increasing concentrations of wild-type DEC1 or 2K/2R and luciferase activity was then determined. (C) Expressions of CLOCK and DEC1 in cells from (B) were detected by western blot with anti-Flag or anti-HA antibody. (D) MCF-7 cells were transfected with HA-tagged wild-type DEC1 or 2K/2R together with Myc-tagged HDAC1. Cell lysate was subjected to IP with anti-HA antibody followed by western blot with anti-Myc antibody. *, *p*<0.05; **, *p*<0.01.

Previous studies have shown that DEC1 acts as a repressor that suppresses CLOCK/BMAL1-mediated activation through binding to E-box [34]. Moreover, SUMOylation is often associated with transcriptional regulation [28], [29]. To examine the impact that SUMOylation of DEC1 has on its repression of CLOCK/BMAL1-mediated transcriptional activity, MCF-7 cells were transiently transfected with a luciferase reporter driven by 3xE-box enhancer elements, and together with Flag-tagged CLOCK, BMAL1 and HA-tagged wild-type DEC1 or its mutant 2K/2R. Luciferase activity assays showed that the extent of repression exerted by the mutant on CLOCK/BMAL1-mediated transcriptional activity was reduced compared to that exerted by wild-type DEC1 (Figure 5A), and the difference was dependent on the gene dosage, but was somewhat increased with increasing dosage of the mutant gene (Figure 5B). This was rather unexpected, but it was repeatedly demonstrated. The expressions of CLOCK and DEC1 were also confirmed by western blot (Figure 5C). Thus SUMOylation of DEC1 was important for its repression of CLOCK/BMAL1 mediated activation in MCF-7 cells.

Repression of DEC1 activity is known to associate with HDAC1 [14]. In order to examine whether SUMOylation would influence the interaction between DEC1 and HDAC1 in the repression of CLOCK/BMAL1-mediated transcriptional activity, MCF-7 cells were transiently transfected with Myc-HDAC1 plus either HA-tagged wild-type or mutant DEC1. The amount of HDAC1 co-precipitated with wild-type DEC1 was about three-fold that co-precipitated with the mutant (Figure 5D), meaning that SUMOylation of DEC1 was important for recruiting HDAC1, which is an essential component for the repression of CLOCK/BMAL1-mediated transcription activity in MCF-7 cells.

### SUMOylation stabilizes DEC1 protein through inhibition of DEC1 ubiquitination

**Figure 6:**
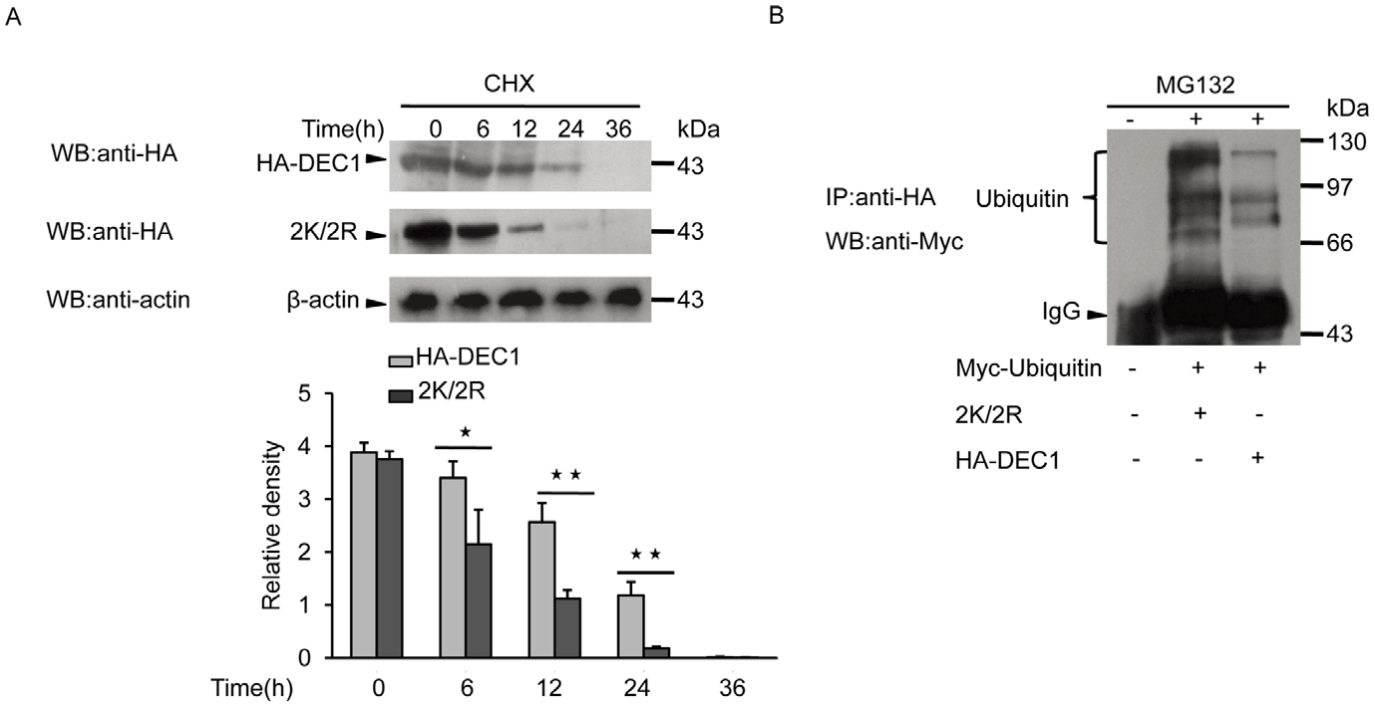
Inhibition of DEC1 ubiquitination by SUMOylation. (A) MCF-7 cells were transfected with HA-tagged wild-type DEC1 or its mutant 2K/2R and treated with CHX at the indicated time periods. Cell lysate was subjected to western blot with anti-HA antibody. (B) MCF-7 cells were transfected with HA-tagged wild-type DEC1 or 2K/2R together with Myc-tagged ubiquitin. Cell lysate was subjected to IP with anti-HA antibody followed by western blot with anti-Myc antibody. *, *p*<0.05; **, *p*<0.01.

The effect of SUMOylation on protein stability was examined by transfecting MCF-7 cells with HA-tagged wild-type or mutant DEC1, followed by treatment of the cells with the protein translation inhibitor cycloheximide (CHX) for five different time periods. The half-life of wild-type DEC1 was between 12 and 24 h, with about 30% of protein remaining at 24 h, whereas the half life of DEC1 mutant 2K/2R was less than 12 h, with only about 5% remaining at 24 h (Figure 6A). This clearly showed that SUMOylation significantly stabilized DEC1 and increased its half-life.

Degradation of DEC1 via the 26 S proteasome pathway requires the covalent attachment of multiple ubiquitin molecules to DEC1. Thus one way in which degradation of DEC1 can be prevented is through inhibiting its ubiquitination. To investigate whether the increased stability of DEC1 resulted from its SUMOylation was a result of suppression of its ubiquitination, MCF-7 cells were transfected with HA-tagged DEC1 mutant 2K/2R and Myc-tagged ubiquitin and the level of DEC1 ubiquitination was compared to that of MCF-7 cells transfected with HA-tagged wild-type DEC1 and Myc-tagged ubiquitin (Figure 6B). Ubiquitination of wild-type DEC1 was reduced compared to the mutant, indicating that SUMOylation of DEC1 inhibited its ubiquitination.

## Discussion

In this report, we demonstrated that the bHLH protein DEC1 can be modified by SUMO1, 2 and 3, which manifested as multiple bands of high molecular weight as seen in SDS-PAGE. These bands were consistent with the presence of multiple SUMOylation sites in DEC1. Previous reports have proposed that many proteins such p53, p68, CTCF and AIB1 are efficiently modified by SUMOs at a lysine residue within the sequence ψKXE [35]–[38]. On the basis of this finding, we identified two major SUMOylation sites, K^159^ and K^279^, in the C-terminus of DEC1. Both sites, which conform to the consensus sequence of ψKXE, can be modified by SUMO, with K^279^ displaying a larger effect, since the K279R mutant showed lesser SUMOylation compared to K159R mutant, although either mutant still retained more than 40% of SUMOylation (relative to wild type) compared to the double mutant. Furthermore, SUMOylation of DEC1 at K^159^ and K^279^ could be reversed by SENP1.

SUMOylation has been shown to regulate the subcellular localization, stability, transcriptional activity and protein-protein interaction of transcription factors [39], [40]. DEC1 is synthesized in the cytoplasm but is then transported into the nucleus where it functions as a transcription factor. DEC1 is transported into the nucleus via the nuclear localization signal-dependent pathway, which traffics a protein into the nucleus via its nuclear localization signal (NLS). NLS binds to a membrane heterodimeric receptor consisting of imporin-α and importin-β subunits, and mediates the subsequent passage of the protein into the nucleus [41]. Computer prediction revealed the presence of a basic NLS at the C-terminus (around residue 286) of DEC1, and replacing K^159^ and K^279^ of DEC1 with arginine somewhat reduced its passage into the nucleus (Figure 4A). The attachment of SUMO to DEC1 may increase the activity of DEC1 NLS, and therefore, enhancing its transportation into the nucleus. This could be an important way to facilitate the function of DEC1 (as a transcription factor).

Hypoxia and the absence of serum are two favorable conditions for tumor cell growth. Response to hypoxia is mediated by the hypoxia-inducible factor 1α (HIF-1α), which is known to regulate the functions of some tumor-associated proteins, such as vascular endothelial growth factor (VEGF), transferrin, and DEC1 [20], [22], [42]. However, the mechanism pertaining to how tumor cells are able to adapt to a serum free condition in vivo (analogous to serum starvation condition in cell culture) at the molecular level is not clear. We showed here that the expression of DEC1 and its SUMOylation were both up-regulated after serum starvation, indicating that the stress of serum starvation may influence the activity of DEC1 through SUMOylation. Since a high level of DEC1 expression has been found in many tumors, we concluded that serum starvation may probably affect the expression and post-translational modification of some tumor-associated proteins in addition to DEC1, leading to tumor proliferation.

As a clock protein, DEC1 is involved in the feed-forward regulation of circadian rhythm. In addition to CLOCK/BMAL1, the circadian clock feedback loop in mammals includes some other clock proteins such as PER, CRY, REV-ERBα and DEC proteins [43], [44]. In this regulatory system, the CLOCK/BMAL1 heterodimer up-regulates other clock genes through direct interaction with the clock elements, including E-box, D-box and ROR/REV-ERB binding elements, whereas DEC, PER and CRY function as repressors of CLOCK/BMAL1-induced transcription by competing with the clock elements for binding to CLOCK or BMAL1 [15], [45]–[48]. Our result showed that SUMOylation of DEC1 led to enhanced repression of CLOCK/BMAL1-mediated transcriptional activity, probably through stronger binding between DEC1 and HDAC1. Binding of DEC1 to HDAC1 may be less stable in the absence of SUMOylation, therefore accounting for a much weaker, but still noticeable repression (∼40%) of CLOCK/BMAL1-mediated transcriptional activity observed in MCF-7 cells transfected with the DEC1 double mutant in which both SUMOylation sites had been abolished (2K/2R, in Figure 5A). In this experiment, the cells were not co-transfected with SUMO1, so the status of DEC1 SUMOylation totally relied on endogenous SUMO activity, and since the DEC1 double mutant 2K/2R could not be SUMOylated, over-expression of this mutant without co-expression of SUMO still justified the conclusion drawn here stating the SUMOylation of DEC1 as part of a regulatory event that governs its interaction with other proteins, such as the transcription factors CLOCK/BMAL1 and the co-repressor HDAC1. The activity of DEC1 may also be regulated by other posttranslational modifications. Nevertheless, our result did suggest that posttranslational modification of DEC1 in the form of SUMOylation may be important, at least, for the regulation of circadian rhythm.

Ubiquitin-dependent proteolysis plays an important role in many basic cellular functions through regulating different cell regulators, such as tumor regulators, transcriptional factors and cell surface receptors [49], [50]. DEC1 is also targeted by ubiquitin [51]. As SUMOylation and ubiquitination are both lysine-targeted modifications, the antagonistic relationship between SUMOylation and ubiquitination may play an important role in regulating DEC1 activity. Interestingly, we found that SUMOylation stabilized DEC1 through inhibiting its ubiquitination, and therefore regulating its activity through enhancing the stability of DEC1. Increase in the stability of DEC1 by SUMOylation was also evident as seen with a drop in the level of nucleolar DEC1 protein with no corresponding increase in its cytoplasmic level (Figure 4A), as well as slight decrease in the level of total DEC1 protein under serum starvation in the case of the DEC1 mutant (Figure 4D).

DEC1 may be considered as a cancer-associated protein, and this is highlighted by its homology with the bHLH domains of hairy protein, which is associated with cell activation and stress in many tissues. However, the molecular mechanisms through which DEC1 may contribute to cancer remain unclear, although its expression has been upregulated in a number of cancers. As posttranslational modifications of proteins are known to play important roles in many cell processes, we speculated that SUMOylation may be one of the mechanisms by which the activity of DEC1 protein is regulated. We showed here that SUMOylation of DEC1 can alter its stability (through reducing its susceptibility to ubiquitination) and enhance its repression of CLOCK/BMAL1 mediated transcriptional activity. However, it is worth noting that other posttranslational modifications of DEC1, such as phosphorylation, acetylation, and methylation have not been well studied, so further studies of the relevance of these posttranslational modifications and their roles in the functions of DEC1 are vital for increasing our understanding of the mechanism by which the activity of DEC1 is regulated, as well as the mechanism by which DEC1 coordinates its activity with other accessory proteins in the regulation of cell processes. Finally, the effect of SUMOlyation on DEC1 target genes will also need to be addressed in order to gain more depth into the importance that it has on DEC1 function, and we are already in the process of carrying out this further investigation.

## Materials and Methods

### Plasmids and antibodies

The Flag-DEC1 construct containing human DEC1 was obtained from Dr K. Tanimoto (Hiroshima University, Japan). Myc-tagged SUMO1, SUMO2 and SUMO3 plasmids were kindly provided by Dr Paul D. Sadowaki (University of Toronto, Canada). The luciferase reporter plasmid containing three clustered E-box elements (CACGTG) cloned into HSV-TK, and the plasmid containing HA-tagged DEC1 were a generous gift from Dr Moritz J. Rossner (Max-Planck-Institute of experimental medicine, Germany). HA-tagged DEC1 mutants (K159R, K279R and K159R/K279R (2K/2R)) were generated using site-directed mutagenesis according to the manufacturer's instructions (Stratagene, La Jolla, CA). Antibody against Flag, Myc and HA (mouse monoclonal and rabbit polyclonal), anti-mouse and anti-rabbit secondary antibodies were purchased from Santa Cruz Biotechnology.

### Cell culture and transfection

COS-7 and MCF-7 cells had been used in our previous study[38]. They were maintained in Dulbecco's modified Eagle's medium (DMEM, Invitrogen) supplemented with 10% fetal bovine serum at 37°C in presence of 5% CO_2_. The cells were transfected using Lipofectamine™ 2000 (Invitrogen) or Vigofect (Vigorous) according to the manufacturer's instructions.

### Immunoprecipitation and western blotting

Cells were seeded at 2×10^5^ per 35-mm dish and cultured for 24 h. The cells were then transfected with 1.5 µg of Flag-tagged wild-type DEC1, HA-tagged wild-type or mutant DEC1 (K159R/K279R) and 2.5 µg of Myc-tagged SUMO1, SUMO2 or SUMO3. After 24 h, cells were lysed in 200 µl buffer containing 50 mM Tris-HCl (pH 8.0), 150 mM NaCl, 0.1% SDS, 1% NP-40 and 0.5% sodium deoxycholate and centrifuged at 10000×*g*/4°C for 10 min. The supernatant was incubated with 2 µg of anti-HA monoclonal antibody for 4 h and then with protein A-Sepharose 4B (Amersham Biosciences) for 12 h at 4°C, followed by centrifugation at 5000×*g*/4°C for 10 min. The pellet was washed twice with wash buffer I (50 mM Tris-HCl, pH 7.5, 500 mM Sodium chloride, 0.1% Nonidet P40, 0.05% Sodium deoxycholate) and once with wash buffer II (50 mM Tris-HCl, pH 7.5, 0.1% Nonidet P40, 0.05% Sodium deoxycholate), and then subjected to SDS-PAGE in 10% gel. Protein bands in the gel were transferred to PVDF membrane and subjected to western blot analysis using the appropriate specific antibody. Immunoblot data were quantified by scanning the appropriate bands of interest and plotted as relative density of gray scale.

### Immunofluorescence staining

MCF-7 cells were cultured for overnight on cover slips immersed in DMEM medium supplemented with 10% fetal bovine serum. The cells were then transfected with HA-tagged wide-type or mutant (K159R/K279R) DEC1. Twenty four hours after transfection the cells were washed three times with PBS, fixed in 1% paraformaldehyde for 15 min at room temperature, permeabilized with methanol for 40 min at −20°C and blocked with 0.8% BSA for 1 h at 4°C. The cells were then incubated with mouse anti-HA monoclonal antibody and examined according to the manufacturer's instructions.

### Luciferase reporter assay

Cells were seeded at 1×10^5^ per well in a 24-well dish and cultured for 24 h before being transfected with appropriate plasmid constructs. Twenty four hours after transfection, the cells were harvested and Luc reporter assay was performed in accordance with the manufacturer's instructions (Promega, USA).

### Statistical Analysis

All statistical analyses of data were preformed with ANOVA with LSD method. Data are given as means ±SDs, and significance was considered at either *P* value <0.05 or 0.01 level.
